# The effect of vaping and smoking on colonic anastomosis healing in an experimental animal model

**DOI:** 10.1038/s41598-026-53666-2

**Published:** 2026-05-19

**Authors:** Mehmet Ali Pak, Fatma Güzel Altıntaş, Göktuğ Okyar, Hikmet Pehlevan Özel, Erdinç Çetinkaya

**Affiliations:** 1 Department of General Surgery, Bingöl Solhan State Hospital, Bingöl, Turkey; 2 Department of Pathology, Ankara Training and Research Hospital, Ankara, Turkey; 3 Department of Biochemistry, Ankara Bilkent City Hospital, Ankara, Turkey; 4 Department of General Surgery, Ankara Bilkent City Hospital, Ankara, Turkey; 5 Department of General Surgery, Hacettepe University, Ankara, Turkey

**Keywords:** Electronic cigarette, Smoking, Colon anastomosis, Wound healing, Experimental animal model, Diseases, Gastroenterology, Health care, Medical research

## Abstract

Anastomotic leakage is a serious complication after colorectal surgery. Smoking is known to impair anastomotic healing. The use of electronic cigarettes is increasing worldwide, and information about their effects on wound healing remains limited. The aim of this study is to investigate and compare the effects of electronic cigarettes and conventional cigarettes on rats. Thirty male Wistar Albino rats were divided into control, smoking, and vaping groups. Exposures were given twice daily for 30 days, and cotinine levels confirmed nicotine intake. On day 30 all animals underwent colonic anastomosis and were sacrificed seven days later. Bursting pressure, histopathology, and hydroxyproline levels were assessed. Cotinine levels were similar in the smoking and vaping groups. Bursting pressure showed no significant differences. Hydroxyproline content and histological scores, however, were significantly reduced in both exposure groups compared with controls, indicating impaired healing. In conclusion, vaping and smoking did not reduce mechanical strength but both impaired collagen synthesis and tissue repair. These results suggest that electronic cigarettes may not be a safer alternative to smoking in the surgical setting.

## Introduction

Colorectal surgery is commonly indicated for malignancies, inflammatory bowel diseases, and mechanical obstructions. Despite significant advancements in surgical techniques and perioperative care, anastomotic leakage remains a serious complication associated with increased morbidity and mortality rates^1^. The healing of a colorectal anastomosis is a complex process influenced by multiple systemic and local factors, including tissue perfusion, the inflammatory response, and collagen synthesis^2^. Among these, cigarette smoking is a well-documented risk factor due to its adverse effects on microvascular circulation, fibroblast activity, and collagen metabolism^3^.

Smoking has long been associated with impaired wound healing, increased oxidative stress, and delayed tissue regeneration^4^. It is a significant risk factor for anastomotic dehiscence, with leakage rates reported to range from 3% to 30% depending on patient- and procedure-related variables^5^. The harmful components of cigarette smoke, including nicotine, carbon monoxide, and various toxins, reduce tissue oxygenation, impair neovascularization, and suppress immune function^6^. In contrast, while the detrimental effects of traditional smoking are well established, the influence of electronic cigarette (e-cigarette) use on tissue repair remains poorly understood and inadequately studied.

E-cigarettes have become increasingly popular, particularly among the younger population, as an alternative to smoking. Marketed as a safer option, e-cigarettes lack combustion and produce fewer toxic byproducts compared to smoking^7^. However, e-cigarette vapor contains nicotine, formaldehyde, acrolein, and other potentially harmful compounds that may affect wound healing^8^. Medical literature on the effects of vaping on gastrointestinal tissue repair is limited, and its safety profile remains controversial. Animal and in vitro studies suggest that vaping may disrupt endothelial function, increase oxidative stress, and impair fibroblast activity, yet the extent of these effects on colorectal anastomosis healing has not been fully elucidated^9^.

Therefore, the present study aimed to evaluate the effects of e-cigarette vapor exposure on the healing of colonic anastomoses in an experimental rat model, using conventional cigarette smoke exposure and non-exposure groups as comparators. The primary hypothesis was that vaping impairs histopathological healing and collagen synthesis to a similar extent as conventional smoking, potentially without significantly affecting the mechanical strength of the anastomosis. Outcome measures included anastomotic bursting pressure, histological healing scores, and hydroxyproline levels as an indicator of collagen content.

## Materials and methods

### Ethical approval

This study was approved by the Animal Experiments Local Ethics and was conducted in full compliance with institutional and international ethical standards for the care and use of laboratory animals. All procedures adhered to the ARRIVE 2.0 guidelines for reporting in vivo animal experiments.

### Study design

This was a controlled, randomized, experimental study designed to evaluate the impact of electronic cigarette vapor on colonic anastomosis healing. The study included three parallel groups (*n* = 10 per group):

- Group I (Control): Underwent colon anastomosis without exposure to cigarette smoke or e-cigarette vapor.
- Group II (Smoking): Exposed to the smoke of five Marlboro Red cigarettes (0.8 mg nicotine, 10 mg tar) twice daily for 30 min for 30 days followed by colon anastomosis.
- Group III (Vaping): Exposed to e-cigarette vapor of Vozol Star 12,000 (Cool Mint, 14 mL liquid container, 50 mg/mL nicotine) in 1-minute intervals over 30 min, twice daily for 30 days at a dose of 160 puffs per session (8 mg/day nicotine) followed by colon anastomosis.

The experimental unit was a single rat. Randomization was performed using a computer-generated random sequence. Investigators responsible for surgical procedures and outcome assessments were blinded to the group assignments.

### Experimental animals

Thirty healthy adults male Wistar Albino rats (age: 8–10 weeks; weight: 200–250 g) were obtained from the Experimental Animal Research Center. Animals were housed in individually ventilated polypropylene cages (3 rats per cage) under standard laboratory conditions (temperature: 22 ± 2 °C, humidity: 50–60%, 12-h light/dark cycle). Standard pellet chow and filtered tap water were provided ad libitum. All animals were acclimatized for 7 days prior to the beginning of the experiment and were confirmed to be free from pathogens. All animals were weighed at the beginning of the experiment, on the day of surgery, and before euthanasia.

### Inclusion and exclusion criteria

All animals underwent pre-procedural health screening. Animals with visible signs of illness, injury, or abnormal behavior were excluded. No animal or data points were excluded from the final analysis. The final sample included all 30 animals (*n* = 10 per group).

### Exposure model

**Fig. 1:**
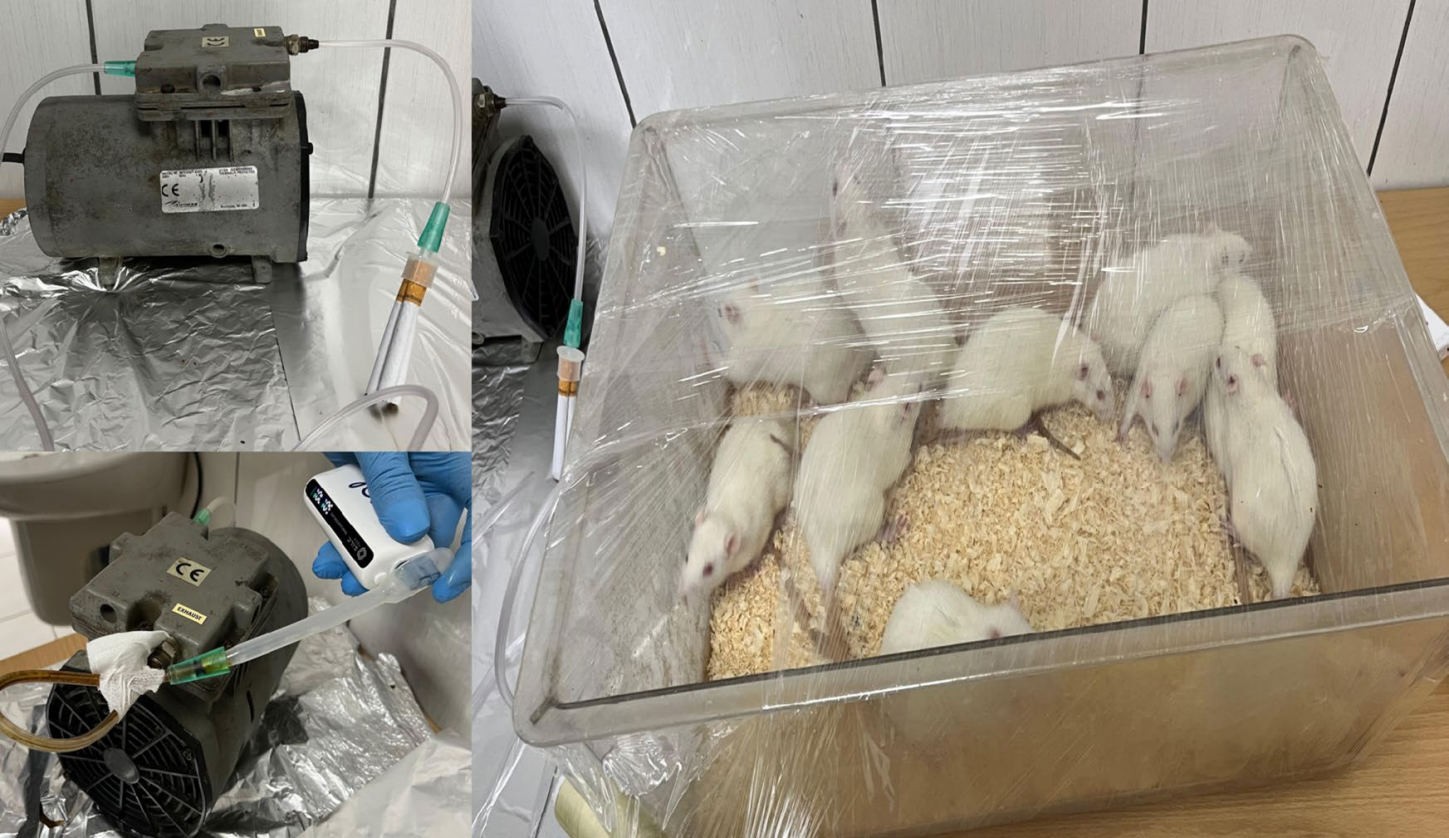
Air pump and exposure chamber.

The cigarette smoke exposure model used in this study was adapted from Karayel et al.^10^. To ensure comparability with previous research, Marlboro Red cigarettes (nicotine: 0.8 mg, tar: 10 mg) were selected based on their use in earlier experimental models^11^. Rats were placed inside a transparent exposure chamber constructed from stretch film with perforations to allow airflow regulation (Fig. 1). A pump-driven system delivered smoke from five cigarettes (nicotine: 0.8 mg, tar: 10 mg) into the chamber during each exposure session. The rats were exposed for 30 min per session, twice daily, at 12-hour intervals, for a total duration of 30 consecutive days.

The electronic cigarette (vaping) exposure model was adapted from Troiano et al.^12^. The device selected for this study was Vozol Star 12,000 (Cool Mint flavor, 14 mL e-liquid capacity, 50 mg/mL nicotine), which is among the most widely used e-cigarette models in Turkey. To simulate real-world inhalation exposure and ensure comparable systemic nicotine levels between groups, nicotine dosing was equated based on estimated absorption.

According to manufacturer data, the device delivers approximately 12,000 puffs per cartridge, with each puff lasting 0.5 s. Based on this, the target daily nicotine dose (approximately 8 mg, equivalent to that received in the cigarette group) was matched to around 160 puffs/day. Each vaping exposure session consisted of four consecutive 10-second puffs, spaced at one-minute intervals, administered twice daily. Animals were exposed via whole-body inhalation in a sealed chamber for 30 min per session, twice daily, over 30 days. Chamber conditions and exposure duration were kept identical between smoking and vaping groups to ensure standardization.

Plasma cotinine levels were measured on days 15 and 30 to confirm systemic nicotine exposure equivalence between groups.

### Plasma cotinine measurement

To confirm nicotine absorption, plasma cotinine levels were measured on days 15 and 30. Blood samples (2 cc) were collected from five randomly selected rats in both the smoking and vaping groups. Consistent with the ethical principle of efficient use of resources additional blood sampling and ELISA analysis were not performed for the control group, as nicotine exposure was not expected. The samples were centrifuged, and plasma cotinine levels were analyzed using an ELISA kit (Cotinine Rat ELISA Kit, SUNRED). This subsample was used for biochemical confirmation of systemic nicotine exposure and not for inferential statistical analysis.

### Surgical procedure

After an 8-hour fasting period, general anesthesia was induced via intramuscular injection of ketamine hydrochloride (10 mg/kg) as the anesthetic agent and xylazine hydrochloride (5 mg/kg) as the analgesic and muscle relaxant. Anesthetic depth was monitored throughout the procedure by evaluating spontaneous respiration and reflex responses.

Once adequate anesthesia was confirmed, the rats were placed in the supine position on a sterile surgical platform. The abdominal fur was shaved, and antisepsis was achieved using 10% povidone-iodine solution. A 4-cm midline laparotomy was performed under sterile conditions, and the peritoneal cavity was accessed by following the anatomical planes.

**Fig. 2:**
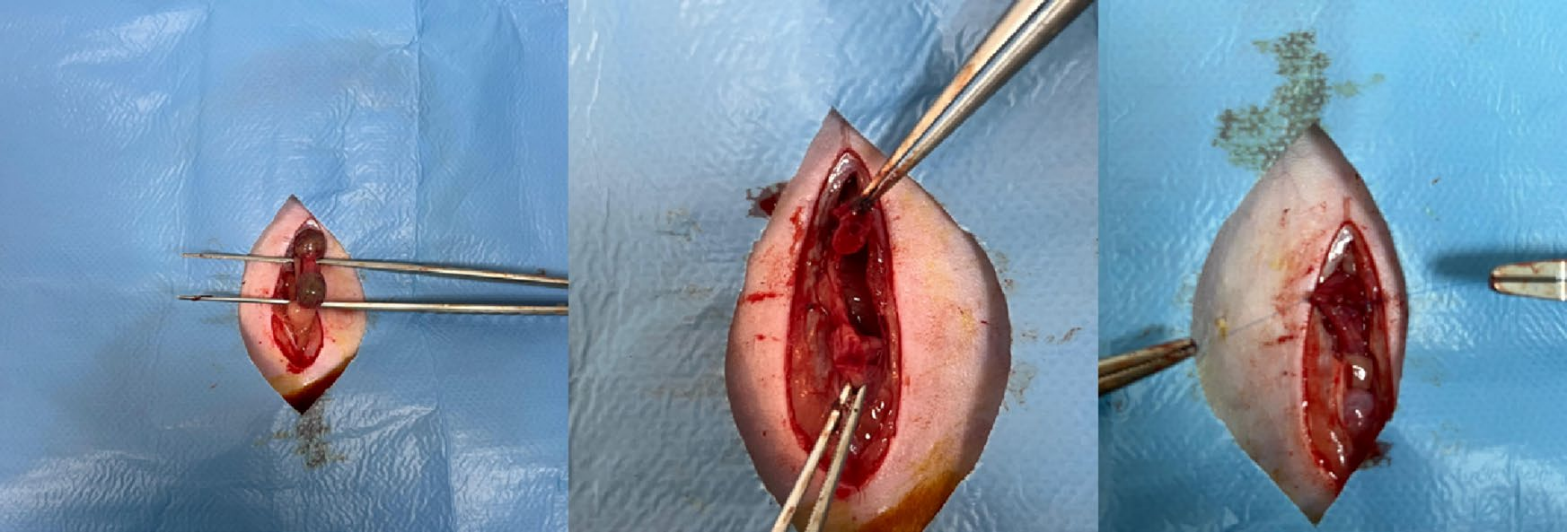
Surgical procedure, end to end anastomosis.

The descending colon was identified, mobilized, and transected. An end-to-end single-layer anastomosis was constructed using eight interrupted simple sutures with 6/0 polypropylene (Fig. 2). After completion of the anastomosis, 10 mL of sterile isotonic saline was administered intraperitoneally for fluid replacement. The abdominal wall and skin were closed in a single continuous layer using 3/0 silk sutures.

Postoperative care included monitoring until full recovery from anesthesia. Rats were allowed access to food and water 6 h after surgery. No intraoperative complications were observed, and all animals survived the surgical procedure.

On postoperative day 7, all rats were anesthetized with an intramuscular injection of ketamine hydrochloride (10 mg/kg) and xylazine hydrochloride (5 mg/kg). Following confirmation of adequate anesthesia, euthanasia was performed using the cervical dislocation method. Subsequently, tissue samples were collected for further histological and biochemical analyses.

### Mechanical assessments

Mechanical evaluations were performed by a single investigator blinded to group allocation to eliminate observer bias.

### Adhesion scoring

On postoperative day 7, animals were sacrificed, and the peritoneal cavity was accessed through a midline incision. The anastomotic site was examined for adhesion formation. Adhesions were scored using the Zühlke Adhesion Scale^13^, which classifies adhesions from grade 0 (no adhesions) to grade 4 (dense, vascularized adhesions requiring sharp dissection). Adhesion severity was recorded before further tissue manipulation.

### Bursting pressure measurement

After adhesion scoring, adhesions around the anastomotic site were carefully separated using blunt or sharp dissection. The anastomotic site was macroscopically evaluated for the presence of abscess formation, dehiscence, and other complications. Subjects with anastomotic dehiscence or leakage were excluded from the bursting pressure measurement to ensure the reliability of the mechanical strength analysis.

**Fig. 3:**
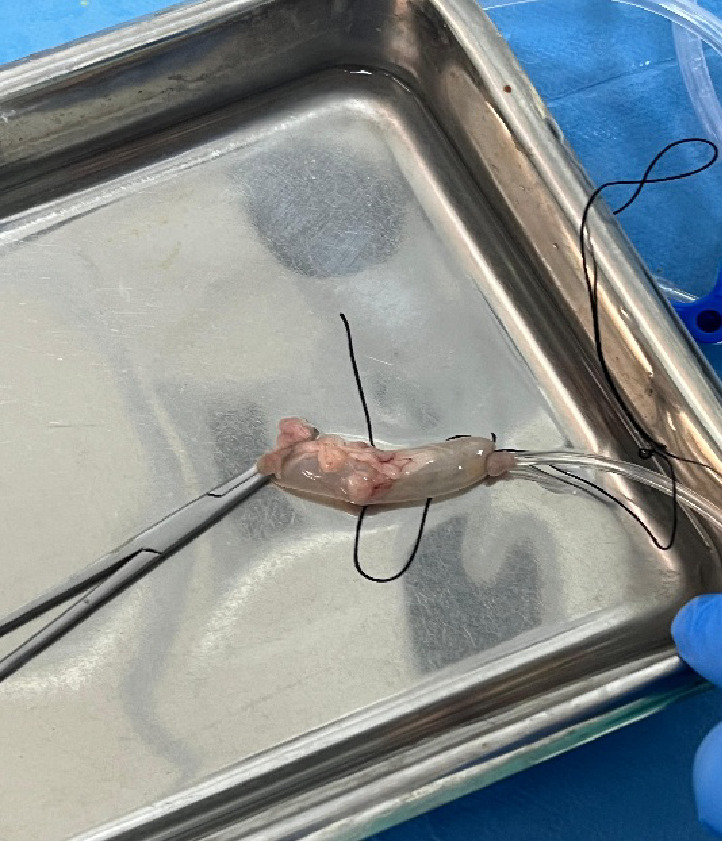
Measuring of bursting pressure.

To assess anastomotic bursting pressure, the colon was transected 2 cm proximally and distal to the anastomotic site, and the excised segment was removed. The distal end of the bowel was clamped, and a pressure catheter connected to a digital manometer was inserted through the proximal end, which was secured with 3/0 silk sutures. The specimen was submerged in a water-filled container, and air was gradually insufflated through the catheter until the first visible air bubbles appeared. The pressure at which this occurred was recorded as the anastomotic bursting pressure and expressed in millimeters of mercury (mmHg) (Fig. 3).

### Histopathological examination

Following the measurement of bursting pressure, each colonic specimen was longitudinally bisected. One half containing the anastomotic site was fixed in 10% neutral-buffered formalin for 48 h. Tissues were then processed using standard histological protocols, embedded in paraffin blocks, sectioned at 4 μm thickness, and stained with hematoxylin and eosin (H&E) and Masson’s Trichrome for collagen evaluation.

A single pathologist, blinded to the group assignments, performed all histopathological examinations using a light microscope. Tissue healing was assessed using the Ehrlich and Hunt Numerical Scale as modified by Philip et al.^14^. This semi-quantitative scoring system evaluates five parameters: inflammatory cell infiltration, neovascularization, fibroblast proliferation, collagen deposition, and re-epithelialization. Each parameter was scored from 0 (none) to 4 (marked), yielding a total possible score of 0 to 20. Higher scores indicated more advanced histological healing.

### Hydroxyproline measurement

A portion of the anastomotic tissue was stored at −80 °C for hydroxyproline analysis. The hydroxyproline content, a marker of collagen deposition, was measured using an ELISA kit (Hydroxyproline Rat ELISA Kit, SUNRED).

### Statistical analysis

The normality of data distribution was assessed using the Shapiro-Wilk test. Continuous variables are expressed as Mean ± SD or Median (Min–Max), depending on the distribution. Group comparisons for bursting pressure were performed using One-Way ANOVA. For histopathological and biochemical parameters, the Kruskal-Wallis test was employed, followed by Bonferroni-adjusted pairwise comparisons for post-hoc analysis. Cotinine levels were compared between groups using the Independent Samples T-test, while the Paired Samples T-test was used to evaluate temporal changes between the 15th and 30th days. To ensure the robustness of the findings, a sensitivity analysis was conducted using the Kruskal-Wallis test. Furthermore, Spearman’s rho correlation analysis was performed to evaluate the associations between histopathological and biochemical variables. A p-value of < 0.05 was considered statistically significant. All statistical analyses were performed using IBM SPSS Statistics 21.0 (IBM Corp., Released 2012, IBM SPSS Statistics for Windows, Version 21.0, Armonk, NY, USA) and MS Excel 2007. A p-value < 0.05 was considered statistically significant.

## Results

The study was completed with 100% survival. However, upon dissection of adhesions, covered anastomotic dehiscence was observed in the smoking (*n* = 2), vaping (*n* = 1), and control (*n* = 1) groups. These cases were recorded as leakage, and because a baseline pressure could not be established, they were omitted from the bursting pressure measurements.

### Changes in body weight over time

During the study period, the initial, preoperative, and before euthanasia (Postoperative day 7) body weights of the rats in each experimental group were evaluated. There were no statistically significant differences in mean body weights among the groups at baseline (*p* = 0.734), preoperative (*p* = 0.166), or postoperative (*p* = 0.092) measurements. This finding indicates that randomization at the beginning of the experiment was successful and that the exposure process did not result in a marked difference in body weight between the groups.

In the time-dependent analysis (repeated-measures ANOVA), a significant change was observed only in the vaping group (F = 6.781; *p* = 0.006). Pairwise comparisons revealed that this change resulted from weight gain between the baseline and preoperative measurements (*p* = 0.020). No significant differences were detected over time in the control or smoking groups (*p* > 0.05). (Table 1).

**Table 1:** Comparison of body weights among groups.

	**Control** **(n=10)**	**Vaping** **(n=10)**	**Smoking** **(n=10)**	**p(Group)**	
	**Mean ± SD** **Median ** **(Min-Max)**	**Mean ± SD** **Median ** **(Min-Max)**	**Mean ± SD** **Median ** **(Min-Max)**		
**Weight**				**2**	
İnitial	223.80 ± 15.43	219.30 ± 12.45	222.50 ± 11.02	F = 0.313	0.734
	222.5 (203–250)	215.5 (202–240)	220.5 (208–240)		
Preoperative	236.10 ± 12.05	250.50 ± 26.54	235.20 ± 17.41	F = 1.918	0.166
	237.5 (218–260)	252.5 (222–308)	236.5 (203–263)		
Postoperative day 7	221.60 ± 19.52	238.60 ± 22.54	221.90 ± 14.07	F = 2.613	0.092
	221.5 (197–267)	242.0 (207–279)	218.5 (202–249)		
**p(Time)**	*F = 2.017; p = 0.162	*F = 6.781; p = **0.006**	*F = 2.451; p = 0.114		

F:One-Way ANOVA Test, *F: Repeated-measures ANOVA test

### Plasma cotinine levels

Plasma cotinine levels were compared between the vaping and smoking groups at the 15th and 30th days. No significant differences were observed between the two groups at either time point (15th day: *p* = 0.950; 30th day: *p* = 0.832).

Within-group comparisons over time revealed that plasma cotinine levels remained stable between the 15th and 30th days in both groups (vaping: *p* = 0.382; smoking: *p* = 0.683), indicating consistent nicotine exposure throughout the study period (Table 2).

**Table 2:** Comparison of plasma cotinine levels between vaping and smoking groups.

	Vaping (*n* = 5)	Smoking (*n* = 5)		
	Mean ± SD	Mean ± SD	*p*(Group)	
**Cotinine plasma** (ng/ml)				
15. Day	172.78 ± 24.23	173.85 ± 28.15	t = 0.065	0.950
30. Day	186.31 ± 17.38	181.49 ± 45.94	t = 0.219	0.832
**p(Time)**	*t = 0.981; *p* = 0.382	*t = 0.440; *p* = 0.683		

*t: Independent Samples T-Test, t: Paired Samples T-Test.

### Adhesion score and bursting pressure

The Adhesion Score did not show significant differences among the groups (*p* = 0.500). The median values were 1.0 (0–4) in the Control group, 0.5 (0–4) in the E-cigarette group, and 1.0 (0–4) in the Cigarette group. Although the Cigarette group had a slightly higher mean score (1.70 ± 1.49) compared to the Control (1.20 ± 1.39) and E-cigarette (1.10 ± 1.45) groups, the differences were not statistically significant.

The rates of anastomotic leakage were 10% (1/10), 20% (2/10), and 10% (1/10) in the control, smoking, and vaping groups, respectively. There was no statistically significant difference between the groups (Fisher’s exact test, *p* = 1.000).

There were no significant differences in anastomotic bursting pressure among the Control, E-cigarette, and Cigarette groups (*p* = 0.970). The median bursting pressure was 220.0 mmHg in all three groups, with interquartile ranges of 140–280 mmHg (Control), 160–260 mmHg (E-cigarette), and 160–280 mmHg (Cigarette). The mean values were also similar (211.67 ± 38.89 mmHg, 215.56 ± 32.06 mmHg, and 215.00 ± 36.64 mmHg, respectively) (Table 2).

**Table 3:** Comparison of bursting pressure (mmHg) between groups.

	**Control **(*n* = 9)	**Vaping **(*n* = 9)	**Smoking **(*n* = 8)	**Test statistic**	
	**Mean ± SD**	**Mean ± SD**	**Mean ± SD**	**F**	**p**
**Bursting pressure** (mmhg)	211.67 ± 38.89	215.56 ± 32.06	215.00 ± 36.64	F = 0.030	0.970

F: One-Way ANOVA Test.

### Hydroxyproline levels

Hydroxyproline levels differed significantly among groups (*p* = 0.002). The Control group had the highest median value (median 1.84, range 1.54–2.48), while both E-cigarette (median 1.19, range 0.87–1.94) and Cigarette groups (median 1.22, range 0.57–1.86) showed significantly lower levels (Table 5).

Pairwise comparisons revealed that both the E-cigarette and Cigarette groups had significantly lower hydroxyproline levels compared to the Control group (*p* = 0.011, *p* = 0.004, respectively), while no significant difference was observed between the E-cigarette and Cigarette groups (*p* = 1.000). These findings suggest that both cigarette and e-cigarette exposure impair collagen synthesis, potentially compromising tissue healing.

**Table 4:** Comparison of hydroxyproline levels between groups.

	Control(n=10)	Vaping(n=10)	Smoking(n=10)	Test statistic	
	Median(Min-Max)	Median(Min-Max)	Median(Min-Max)	x^2^	p
Hydroxyproline (microgram/mg protein)	1.84 (1.54–2.48.54.48)	1.19 (0.87–1.94.87.94)	1.22 (0.57–1.86.57.86)	x^2^ = 12.483	0.002

χ^2^:Kruskal Wallis Test.

### Histopathologic assessment

**Fig. 4:**
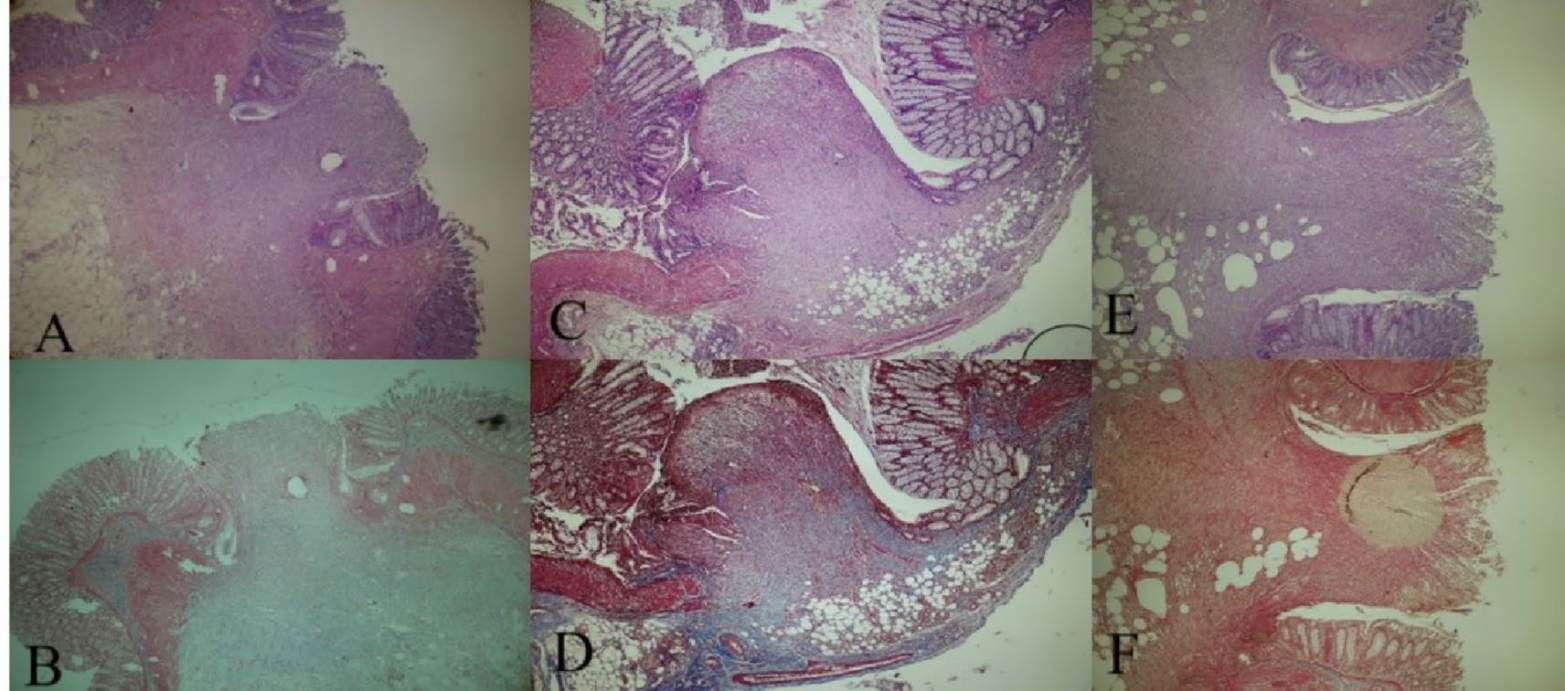
**(A-C-E)** Control, Smoking, and Vaping groups stained with Haematoxylin-Eosin (**H&E**) at 40× magnification (**B-D-F)** Control, Smoking, and Vaping groups stained with Masson Trichrome at 40× magnification.

Histopathologic score (Philip et al. Modified Ehrlich and Hunt Numerical Scale), representing overall histopathological healing, was significantly lower in both the E-cigarette (9.20 ± 1.23, median 9.5, *p* < 0.001) and Cigarette groups (10.20 ± 3.29, median 10.5, *p* < 0.001) compared to the Control group (16.50 ± 2.63, median 17.0) (Table 4) (Fig. 4).

Pairwise comparisons showed that E-cigarette vs. Control (*p* < 0.001) and Cigarette vs. Control (*p* = 0.008) differences were significant, while E-cigarette vs. Cigarette (*p* = 1.000) was not. These findings suggest that both cigarette and e-cigarette exposure significantly impair tissue healing compared to the control group, with no significant difference between the exposure groups.

**Table 5:** Comparison of histopathological score values by groups.

	Control (n=10)	Vaping (n=10)	Smoking (n=10)		
	Median (Min-Max)	Median (Min-Max)	Median (Min-Max)	x^2^	p
İnflammatory cell infiltration	4.0 (3–4)	2.0 (1–2)	2.0 (1–3)	x^2^ = 19.135	<0.001
Fibroblast	3.0 (2–4)	2.0 (2–3)	2.0 (1–3)	x^2^ = 6.972	0.031
Neovascularisation	3.0 (2–4)	2.0 (1–2)	2.0 (1–3)	x^2^ = 14.345	0.001
Collagen deposition	3.5 (2–4)	2.0 (1–3)	2.0 (1–3)	x^2^ = 14.549	0.001
Reepithelization	3.0 (0–4)	2.0 (0–3)	2.0 (0–3)	x^2^ = 8.584	0.014
Total score	17.0 (10–19)	9.5 (7–11)	10.5 (4–15)	x^2^ = 17.028	<0.0:01

x^2^ :Kruskal Wallis Test.

### Sensitivity analysis

A sensitivity analysis conducted by excluding subjects with anastomotic leakage revealed that the statistical significance among histopathological scores remained robust (Kruskal–Wallis χ² = 19.293, *p* < 0.001).

### Correlation analysis

In the correlation analysis, a strong positive relationship was identified between hydroxyproline levels and histopathological scores (ρ = 0.874, *p* < 0.001). A moderate positive correlation was found between hydroxyproline and bursting pressure (ρ = 0.619, *p* = 0.001). In contrast, the association between histopathological scores and bursting pressure did not reach statistical significance (ρ = 0.380, *p* = 0.055).

### Relationship between anastomotic leakage and healing parameters

When comparing animals that developed anastomotic leakage with those that did not, both total histopathological scores and hydroxyproline levels were found to be significantly lower in the group that developed leakage (Mann–Whitney U = 8.0, *p* = 0.007 and U = 11.0, *p* = 0.012, respectively).

## Discussion

Anastomotic leakage remains one of the most critical complications following colorectal surgery, substantially contributing to increased healthcare expenditures, postoperative morbidity, and mortality rates^15^. Over the past century, extensive research has been devoted to elucidating the underlying mechanisms of anastomotic failure, identifying contributing risk factors, and developing preventive strategies^16^. Among these, cigarette smoking has been consistently identified as a significant risk factor, with numerous studies substantiating its detrimental impact on anastomotic healing^17^. In contrast, e-cigarettes, which have gained popularity in the past 15 years, represent a relatively novel exposure type, and their effects on gastrointestinal physiology and wound healing remain incompletely understood. Current literature addressing the potential impact of e-cigarette use on the gastrointestinal system is limited. The present investigation was conducted to evaluate the effects of e-cigarette vapor on gastrointestinal wound healing and colonic anastomotic integrity.

In this experimental study, the effects of electronic cigarette (vaping) and conventional cigarette exposure on colonic anastomotic healing were evaluated through a multidimensional approach. Our findings demonstrate that both types of exposure significantly compromise the biological components of anastomotic repair; however, mechanical strength remains preserved during the early postoperative phase. This apparent dissociation between mechanical integrity and biological healing represents a central finding of the present stud. Crucially, the observed disruptions in histopathological healing and collagen synthesis were found to be directly associated with the occurrence of anastomotic fragility In this context, we define a “biologically fragile anastomosis” as a condition characterized by reduced collagen synthesis and impaired histological maturation despite preserved mechanical strength. Rather than indicating overt clinical failure, these findings should be interpreted as evidence of early microstructural impairment that may compromise tissue resilience under physiological stress conditions.

A critical finding of our study is the discrepancy between mechanical strength (bursting pressure) and biochemical/histological recovery. Although bursting pressures remained statistically similar across groups, the significant decline in hydroxyproline levels and histological maturation scores indicates a ‘biologically fragile’ anastomosis. In the early phase of anastomotic healing (day 7), mechanical resistance is often maintained by peri-anastomotic adhesions and the initial stabilization of the collagen matrix. However, as Koruda et al. noted, true anastomotic strength ultimately depends on the quality and cross-linking of submucosal collagen^18^, not just the absolute pressure required for disruption. Accordingly, the reduced hydroxyproline levels observed in both the e-cigarette and smoking groups suggest that, despite preserved short-term mechanical resistance, the underlying regenerative capacity of the anastomosis is compromised at a microstructural level. This finding should be interpreted cautiously and may reflect a biological vulnerability rather than established clinical leakage.

The absence of significant differences in histopathological scores and hydroxyproline levels between the smoking and e-cigarette groups indicates that both exposures exert comparable detrimental effects on tissue healing. This observation challenges the perception that e-cigarettes represent a safer alternative in the perioperative setting. Our findings align with literature linking e-cigarette exposure to oxidative stress, endothelial dysfunction, and inflammation, particularly regarding impaired collagen synthesis. Although individual-level correlation analysis was not feasible due to limited cotinine sampling, comparable group-level cotinine concentrations support the validity and equivalence of nicotine exposure between groups. Future research should further investigate the specific relationship between individual exposure levels and healing outcomes.

Although numerous in vitro and in vivo experimental studies have explored the effects of e-cigarettes, relatively few have focused specifically on wound healing within the gastrointestinal system. Djouina et al. investigated the long-term pathological consequences of e-cigarette exposure in an animal model, demonstrating that six months of chronic exposure impaired epithelial renewal, immune function, and barrier integrity in the colon and terminal ileum. Both cigarette smoke and e-cigarette vapor were shown to disrupt epithelial renewal in the ileum and upregulate cyclin D1 expression. Notably, e-cigarette exposure resulted in reduced cellular proliferation in the colon, a finding not observed with conventional cigarette smoke. Furthermore, both types of exposure were associated with decreased expression of tight junction proteins and significant alterations in gut microbiota composition. Mucosal immune responses, particularly in the colon, were substantially impaired, with e-cigarette effects reported to be comparable to those of conventional smoking^19^. Sharma et al. similarly demonstrated that e-cigarette exposure increased intestinal permeability and disrupted immune regulatory mechanisms^20^. In the present study, histopathological analysis revealed attenuated inflammatory cell infiltration and diminished reepithelialisation, consistent with the findings reported in the literature.

Heba et al. conducted an animal study evaluating oxidative and inflammatory responses in the colonic mucosa following short-term (4-week) exposure to e-cigarette vapor. The results demonstrated significantly elevated oxidative stress markers, including malondialdehyde and nitric oxide, as well as increased tumor necrosis factor-alpha (TNF-α) levels, accompanied by a reduction in antioxidant markers. These alterations in immune response and redox balance were associated with impaired mucosal regeneration and a decrease in goblet cell density^9^. Consistent with these findings, the present study also identified a marked reduction in fibroblast activity and collagen deposition in the e-cigarette exposure group, suggesting a similarly adverse impact on mucosal repair. Collectively, these studies support a converging mechanism of impaired epithelial regeneration and barrier dysfunction associated with e-cigarette exposure.

Beyond the gastrointestinal system, several studies have investigated the effects of e-cigarette exposure on general wound healing. Liu et al. demonstrated that e-cigarette vapor induces endothelial apoptosis and compromises vascular function. Both in vitro and in vivo experiments revealed delayed wound healing in diabetic rats following e-cigarette exposure. Supporting these findings, Jaleel et al. reported similar vascular impairments, indicating that e-cigarettes adversely affect endothelial function through analogous mechanisms^21^. Rau et al. further demonstrated that e-cigarette vapor impairs skin graft viability to a degree comparable with conventional cigarette smoke^22^, while Kennedy et al. found that tendon healing was equally compromised by both exposure types^23^. Taken together, these findings indicate that the detrimental effects of e-cigarette exposure on wound healing are systemic and not limited to the gastrointestinal tract.

Consistent with the findings of Ashour et al., our results underscore that e-cigarette vapor should not be perceived as a safer perioperative alternative, as it induces systemic and cellular impairments comparable to traditional smoking. Despite preserved mechanical strength, the observed reduction in hydroxyproline levels and fibroblast activity supports the presence of a biologically compromised anastomosis that may be predisposed to delayed or stress-induced failure. While we observed maintained mechanical bursting pressures, the significant reduction in hydroxyproline levels and fibroblast proliferation suggests a ‘biologically fragile’ anastomosis, potentially predisposed to delayed complications. This biological decline aligns with evidence from systematic reviews indicating that e-cigarette constituents exacerbate tissue hypoxia through nicotine-induced catecholamine release and thromboxane A2-mediated vasoconstriction, while simultaneously inhibiting fibroblast differentiation via TGF-β suppression^24^. Furthermore, the equivalence of plasma cotinine levels between exposure groups confirms that e-cigarette use delivers a systemic nicotine burden comparable to conventional smoking, reinforcing the clinical relevance of these findings.

A systematic review by Bulantrisna et al. concluded that e-cigarettes negatively influence wound healing via multiple mechanisms, including osmotic imbalance, cellular toxicity, vasoconstriction, impaired oxygen delivery, disrupted angiogenesis, and altered inflammatory responses. Nonetheless, these effects were reported to be less severe than those associated with traditional cigarette use^25^. In contrast, Philips et al. evaluated the toxicity of key e-cigarette components and found that propylene glycol (PG) and vegetable glycerin (VG), in the absence of nicotine or flavoring agents, did not cause systemic toxicity^26^. These findings suggest that nicotine and flavoring agents may be the primary drivers of the adverse biological effects associated with e-cigarette exposure.

This study has several limitations that should be acknowledged when interpreting the findings. First, the use of an experimental animal model introduces inherent limitations in terms of extrapolating the results to human physiology. Furthermore, while the 7-day follow-up period employed in this study is standard for assessing early healing phases, it may not capture potential late-term complications. Moreover only male rats were included to eliminate the potential influence of hormonal variability; thus, the applicability of the findings to female populations may be restricted. Although the cigarette and electronic cigarette exposure protocols were adapted from previously published experimental models, the exposure conditions used in this study may not fully replicate human smoking and vaping behaviors, thereby limiting the generalizability of the findings to clinical settings.

Another notable limitation pertains to the lack of standardization among e-cigarette products, which complicates comparisons across studies. With over 7,000 e-cigarette devices and e-liquid formulations currently available on the global market^27^, variations in device specifications and flavoring agents present significant challenges to standardization. In alignment with other studies, the most frequently used e-cigarette model in the relevant geographic region was selected. Nevertheless, the exposure method—whole-body inhalation within a sealed chamber—may differ from real-life usage patterns. For comparison, Djouina et al. implemented a nasal inhalation model that allowed for more controlled delivery. The extent to which cutaneous absorption of e-cigarette vapor contributes to systemic effects remains unclear but is theoretically possible^19^.

The exposure model in this study was based on manual administration of e-cigarette vapor. In contrast, other studies have implemented diverse methodologies to standardize exposure conditions, including the use of oxygen-blended vapor delivery systems^23^, automated smoking machines^22^, and nasal airflow devices^26^. Verification of nicotine exposure is frequently achieved through the measurement of cotinine, a primary nicotine metabolite^22,23,25^, while carbon monoxide levels have also been utilized as supplementary validation parameters^19^. In this study, plasma cotinine concentrations measured on days 15 and 30 were comparable to levels reported in active cigarette smokers, supporting the reliability and systemic equivalence of the exposure model.

A comprehensive review of the literature (PubMed–Google Scholar) did not identify any previous experimental or clinical studies specifically evaluating the effects of e-cigarette exposure on colonic anastomosis healing. Nevertheless, the present findings are consistent with existing evidence on wound healing and gastrointestinal integrity in the context of nicotine exposure. Given the rising global prevalence of e-cigarette use, concerns persist regarding their potential to cause harm similar to conventional smoking^28^. These concerns underscore the necessity for governmental regulation to ensure transparency regarding the constituents of e-cigarette products and their potential health implications^29^. Continued clinical and experimental investigations are warranted to elucidate the long-term effects of e-cigarette use on surgical and gastrointestinal outcomes.

## Conclusion

Vaping and smoking do not significantly alter the mechanical integrity of colonic anastomoses in this experimental model, despite inducing significant histopathological alterations and reducing collagen synthesis. These findings suggest that e-cigarette use may represent a risk factor comparable to conventional smoking in the perioperative setting. Further studies with larger sample sizes and extended follow-up are required to determine the clinical implications of these findings.

## Data Availability

The data reported in this study can be obtained from the corresponding author upon reasonable request.

## Declarations

### Institutional animal care and use committee

Animal experiment study was approved by the Animal Experiments Local Ethics Committee (Approval No: 0081, dated 21 March 2024). The original version of the ethics committee approval has been uploaded under the “Related Files” section.

### ARRIVE guidelines

The authors have read the ARRIVE guidelines, and the manuscript was prepared and revised according to the ARRIVE guidelines.
